# Spatio-Temporal Analysis of Leptospirosis Hotspot Areas and Its Association With Hydroclimatic Factors in Selangor, Malaysia: Protocol for an Ecological Cross-sectional Study

**DOI:** 10.2196/43712

**Published:** 2023-05-15

**Authors:** Muhammad Akram Ab Kadir, Rosliza Abdul Manaf, Siti Aisah Mokhtar, Luthffi Idzhar Ismail

**Affiliations:** 1 Department of Community Health Faculty of Medicine and Health Sciences Universiti Putra Malaysia Selangor Malaysia; 2 Department of Electrical & Electronic Engineering Faculty of Engineering Universiti Putra Malaysia Selangor Malaysia

**Keywords:** leptospirosis, hotspot areas, hydroclimatic factors, Selangor, geographical information system, GIS, predictive model

## Abstract

**Background:**

Leptospirosis is considered a neglected zoonotic disease in temperate regions but an endemic disease in countries with tropical climates such as South America, Southern Asia, and Southeast Asia. There has been an increase in leptospirosis incidence in Malaysia from 1.45 to 25.94 cases per 100,000 population between 2005 and 2014. With increasing incidence in Selangor, Malaysia, and frequent climate change dynamics, a study on the disease hotspot areas and their association with the hydroclimatic factors would further enhance disease surveillance and public health interventions.

**Objective:**

This study aims to examine the association between the spatio-temporal distribution of leptospirosis hotspot areas from 2011 to 2019 with the hydroclimatic factors in Selangor using the geographical information system and remote sensing techniques to develop a leptospirosis hotspot predictive model.

**Methods:**

This will be an ecological cross-sectional study with geographical information system and remote sensing mapping and analysis concerning leptospirosis using secondary data. Leptospirosis cases in Selangor from January 2011 to December 2019 shall be obtained from the Selangor State Health Department. Laboratory-confirmed cases with data on the possible source of infection would be identified and georeferenced according to their longitude and latitudes. Topographic data consisting of subdistrict boundaries and the distribution of rivers in Selangor will be obtained from the Department of Survey and Mapping. The ArcGIS Pro software will be used to evaluate the clustering of the cases and mapped using the Getis-Ord Gi* tool. The satellite images for rainfall and land surface temperature will be acquired from the Giovanni National Aeronautics and Space Administration EarthData website and processed to obtain the average monthly values in millimeters and degrees Celsius. Meanwhile, the average monthly river hydrometric levels will be obtained from the Department of Drainage and Irrigation. Data are then inputted as thematic layers and in the ArcGIS software for further analysis. The artificial neural network analysis in artificial intelligence Phyton software will then be used to obtain the leptospirosis hotspot predictive model.

**Results:**

This research was funded as of November 2022. Data collection, processing, and analysis commenced in December 2022, and the results of the study are expected to be published by the end of 2024. The leptospirosis distribution and clusters may be significantly associated with the hydroclimatic factors of rainfall, land surface temperature, and the river hydrometric level.

**Conclusions:**

This study will explore the associations of leptospirosis hotspot areas with the hydroclimatic factors in Selangor and subsequently the development of a leptospirosis predictive model. The constructed predictive model could potentially be used to design and enhance public health initiatives for disease prevention.

**International Registered Report Identifier (IRRID):**

PRR1-10.2196/43712

## Introduction

### Background

Leptospirosis is a neglected zoonotic disease caused by the bacteria agent from the genus *Leptospira* [1,2]. The disease primarily affects vulnerable populations in the urban slums, especially in developing countries, rural farmers, and those involved with water recreational activities [3,4]. Outbreaks are also reported during heavy rainfall and flood seasons and in areas with higher humidity and temperature climates [5].

Although animals are the primary reservoir of *Leptospira*, a human could acquire the infection through the percutaneous contact of contaminated water or soil with the urine of infected animals [1,6]. The clinical presentation could range from afebrile flu-like symptoms to severe manifestations such as pneumonia, acute kidney injury, pulmonary hemorrhages, and even death. Although most infection causes are mild symptoms and can resolve without requiring treatment or be settled with antibiotics, the spread of infection is best controlled by applying preventive measures to minimize potential outbreaks and mortality [6]. Furthermore, better surveillance and disease control could be achieved by integrating hydroclimatic factors of the disease with spatio-temporal hotspot area analysis [7].

Based on morbidity and mortality from hospital-based surveillance studies, the global burden of leptospirosis was estimated to be 1.03 million cases, with 58,900 deaths per year [3,8]. From these data, the disease burden in terms of disability-adjusted life years was 2.90 million per annum, translating to 2.80 million years of life lost and 103,200 years lived with a disability [3]. The Leptospirosis Epidemiology Reference Group of the World Health Organization was established in 2009 to estimate the global burden of leptospirosis. According to the Leptospirosis Epidemiology Reference Group report in 2011 summarizing studies on leptospirosis complications and case fatality in a systematic review, there were 36% (range 0%-88%) of patients reported having an acute renal injury, while 17% reported acquiring acute lung injury (range 0%-62%). Among them, 12% and 25% had died from disease complications, respectively [8]. Pulmonary hemorrhage syndrome is another severe manifestation of leptospirosis that could be life threatening [8].

Leptospirosis is endemic in humid and tropical regions in South Asia, Southeast Asia, and Southern America. Developed countries in temperate zones, such as in the United States and Europe, report fewer cases due to unfavorable environmental conditions for the bacteria’s animal reservoirs, thus considering the disease neglected [9]. However, outbreaks in the aforementioned regions have been reported as having been linked to travel-related reasons to endemic countries or to water activities [1,6].

The disease occurs more in suitable environments with poor sanitation and hygiene conditions, favoring the survival and proliferation of the rodent population, the leading animal reservoir in the South East Asia (SEA) region. [9]. The SEA countries are in the tropical belt, which experiences warm to hot climates with high rainfalls during wet seasons. This phenomenon promotes soil moisture, favoring *Leptospira* growth, subsequent contact, and transmission to humans [10]. An epidemiological study on the agent for human leptospirosis prevalent in the SEA countries identified the main *Leptospira* species pathogenic to humans; *L. interrogans* and *L. borgpeterseni* [11].

A meta-analysis studying the risk factors for leptospirosis following flooding discovered that the presence of rodents squirming near human settlements following the catastrophe and adult males having abrasion wounds or walking barefoot were significantly associated with the infection [12]. For instance, a massive flood following heavy rainfall hit Kerala, India, which took around 500 lives, and approximately 70 were reportedly killed by leptospirosis or dengue fever [13]. Indonesia, a country in the SEA region in the tropical rain belt, has seen 122 deaths from 920 leptospirosis cases reported in 2019 [14,15]. The country often experiences floods that commonly act as a catalyst for stagnant water, and poor sewer and sanitation systems are risk factors for leptospirosis infection [15].

Malaysia has the climate of a dynamically alternating, moderately hot and humid environment throughout the year, with bouts of heavy rains that could cause floods in prone regions. These characteristics are known to influence regional leptospirosis occurrence in Argentina, where the monthly precipitation, monthly river hydrometric level, and Oceanic Nino Index were the hydroclimatic indicators of the disease [16]. On the other hand, a study in Africa demonstrated that rainfall influences the population of rodents, which act as vectors for leptospirosis [17].

Leptospirosis is endemic in Malaysia, affecting mainly 5 states from 2005 to 2014, namely Kelantan, Selangor, Sarawak, Sabah, and Perak. The incidence in these states was constantly increasing and caused a peak of cases in 2014 [18]. Significant leptospirosis outbreaks have occurred on several occasions, such as during an Eco-Challenge in Sabah in 2000 and following a major flood event that mainly hit the northeastern states of Malaysia in 2014 [19,20]. Out of the 304 athletes who participated in the adventure sport in Borneo, 80 had met the clinical case definition of leptospirosis, with 26 of them testing positive for leptospirosis immunoglobulin M by enzyme-linked immunosorbent assay [19]. Meanwhile, Kelantan, one of the states hit by a major flood in 2014, saw more than 1000 leptospirosis cases identified during and after the flood incident [20].

Selangor is one of the states facing a high incidence of leptospirosis. The state recorded the highest cases in 2013, with the highest incidence rate of 24.68 per 100,000 population [21]. Even though there has been a steady rise in reported leptospirosis cases, the actual incidence might be underestimated as the clinical symptoms resemble other endemic acute febrile illnesses such as dengue fever and malaria [22]. The impacts of climate change with increased flood intensities and frequent weather fluctuations increase vector population and enhance pathogenic *Leptospira* growth [23].

The geographic information system is a tool to analyze disease patterns through techniques such as spatial and temporal analysis [24,25]. A spatio-temporal study combines geographic data as well as trends of disease patterns over a specified period, subsequently generating a predictive model for disease management [25,26]. Walter Langbein, a Scientific Hydrologist, defines hydroclimatology as the “study of the influence of climate upon the waters of the land” [27]. The study of hydroclimatic factors concerned with leptospirosis infection would include factors of temperature, precipitation events, watercourses such as rivers and streams, and floods [16,28].

### Objectives

This research aims to explore the spatio-temporal distribution of leptospirosis hotspot areas and their association with the hydroclimatic factors to develop a predictive hotspot area using geographic information system and remote sensing methods. Therefore, this study conducted in Selangor would be able to (1) describe the characteristics of leptospirosis cases in Selangor from 2011 to 2019; (2) map the spatio-temporal distribution of the leptospirosis cases; (3) develop leptospirosis hotspot maps; (4) explore the hydroclimatic characteristics (monthly rainfall, monthly river hydrometric level, and monthly land surface temperature) for Selangor; (5) examine the association between hydroclimatic factors and leptospirosis hotspot areas; and (6) develop a leptospirosis hotspot predictive model based on the hydroclimatic factors.

This potentially life-threatening condition will be challenging to address if potential outbreaks are not predicted with hotspot areas not being outlined. Furthermore, this research may act as a pilot study in the country for the spatio-temporal analysis of leptospirosis with its association with hydroclimatic factors. The predictive model produced in this study can be replicated for future applications and studies.

## Methods

### Regional Setting

#### Study Location

The state of Selangor is located in the central part of the west coast of Peninsular Malaysia, bordered to the North, East, and South by Perak, Pahang, and Negeri Sembilan. Selangor lies to the west of the Titiwangsa range between the latitudes of 2°39’18.0” and 3°50’54.4” north to the equator and longitudes of 101°44’49.0” and 100°52’07.7” east. The state covers an area of approximately 8100 km^2^. There are 55 subdistricts, also known as mukim, in 9 districts within Selangor (ie, Sabak Bernam, Hulu Selangor, Kuala Selangor, Gombak, Klang, Petaling, Hulu Langat, Kuala Langat, and Sepang). Generally, the irrigation in Selangor comes from 4 main river basins, namely Sungai Selangor and Sungai Bernam to the north, and Sungai Langat and Sungai Klang to the south. Historically, these major rivers were seen as the major drivers of early community settlements around these areas.

#### Study Design

This will be a retrospective, hydroclimatic study concerning leptospirosis, whereby secondary data of leptospirosis cases and hydroclimatic factors (rainfall, land surface temperature [LST], and river hydrometric data) will be obtained over a period of 10 years, from January 2011 to December 2019.

#### Study Population

The study population of this study will be leptospirosis cases in the Selangor state from January 2011 to December 2019.

#### Sampling Method

The sample population includes all mukim polygons in Selangor. The sampling frame will be the lists of mukim areas in Selangor, with each polygon representing a sampling unit. The sampling size of the study will be 54 mukim polygons in Selangor using a universal sampling method of notified leptospirosis cases.

### Data Collection

Data on leptospirosis cases from 2011 to 2019 will be obtained from the Zoonosis Unit, Selangor State Health Department. A period of 9 years was taken to compare the results from other similar studies in Argentina and Malaysia [16,29]. The data obtained will be analyzed for the sociodemographic information of the cases and locations of possible sources of infection with their longitude and latitude coordinates. Permission for data collection from relevant agencies has been obtained from the Medical Research and Ethics Committee, the Ministry of Health, and the Ethics Committee for Research Involving Human Subjects before the commencement of the study.

The topographical data for the Selangor state will be obtained from the Department of Survey and Mapping (JUPEM). The data will be in the shapefile format. Satellite images from the Integrated Multi-satellite Retrievals for Global Precipitation Management will be used to derive rainfall data for the Selangor state from January 1, 2011, to December 31, 2019. Images will be obtained from the Giovanni NASA EarthData website [30]. The LST is measured using the MOD11A2 satellite through the onboard Moderate Resolution Imaging Spectroradiometer sensor. It captures 1 km/pixel images at an emissivity of 8 days. Data will be downloaded in a Network Common Data Form format [30]. Meanwhile, the river hydrometric level of rivers in Selangor will be obtained from the Department of Drainage and Irrigation (DID) over a period from January 1, 2011, to December 31, 2019.

### Operational Definition of Variables

#### Dependent Variable

Leptospirosis hotspot area is defined as zones that are hydroclimatically hotspots for leptospirosis transmission in Selangor. The zones will be divided into buffer zones according to color coding based on susceptibility, which are as follows: (1) green zone, not susceptible; (2) yellow zone, less susceptible; (3) orange zone, susceptible; and (4) red zone, highly susceptible.

#### Independent Variables

**Figure 1:**
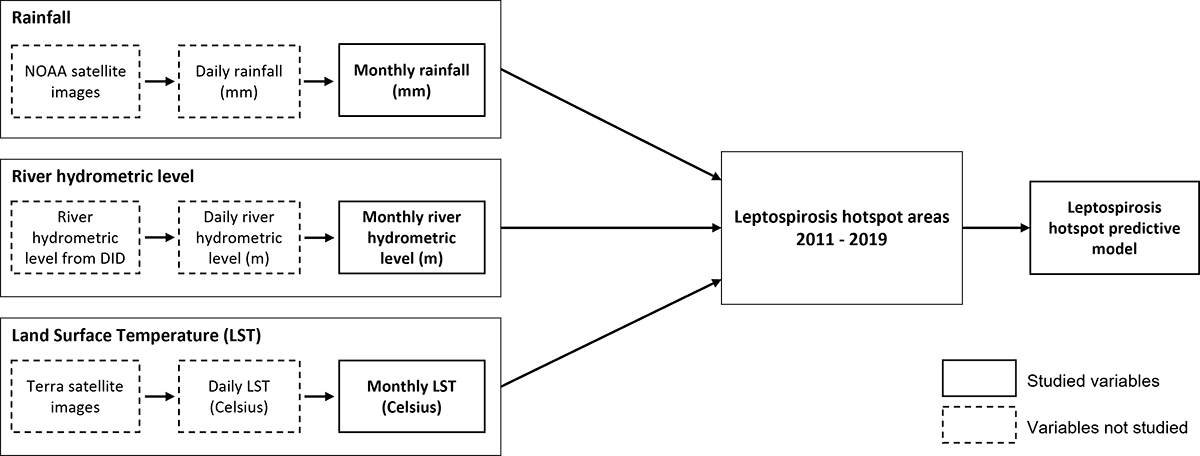
Conceptual framework. DID: Department of Drainage and Irrigation; LST: land surface temperature; NOAA: National Oceanic and Atmospheric Administration.

The independent variables for the study are as follows: (1) the rainfall images in millimeters captured by the satellite will be analyzed to obtain the average monthly rainfall; (2) the LST is the earth’s surface temperature captured by the satellite in degrees Celsius; and (3) the river hydrometric level is the water level threshold classified for risk of flooding according to the DID, into normal, alert, warning and danger. The alert level is when the level increases significantly above average. In the warning stage, the level increases to near flooding, and preparation for evacuation is commenced. When the level reaches the danger level, a significant flooding event would likely occur, and the evacuation of people is initiated. Figure 1 shows the conceptual framework of this study. The framework outlines the geospatial processes of satellite photography images and river hydrometric level and their association with leptospirosis hotspot areas in Selangor from 2011 to 2019.

### Data Processing

#### Processing Leptospirosis Data

The data cases will be cleaned and mapped according to the spatio-temporal variability to identify areas and periods with the highest incidence. The longitudes and latitudes of the possible source of infection of cases will be determined and reconfirmed with JUPEM. Subsequently, the coordinate table in Excel (Microsoft Corporation) will be added in the CSV format and imported to the ArcGIS Pro software (Esri) to determine the spatial distribution of leptospirosis cases. After analysis with the independent variables, leptospirosis hotspot areas will be generated and clipped with mukim polygon areas in Selangor.

#### Processing Topographical Data

The shapefile obtained from JUPEM will be projected in ArcGIS Pro software and extracted to thematic layers. The river hydrometric level data will be averaged into monthly data, tabled in Excel, and then saved in the CSV file format. Zonal statistics will be used for the analysis of each polygon.

#### Processing Satellite Data

Images obtained will be processed to obtain average monthly data for rainfall and LST for each mukim using the clipping tool in the ArcGIS Pro software to extract temperature and rainfall data for each polygon.

### Data Analysis

The descriptive statistics of leptospirosis cases for each month from 2011 to 2019 will be analyzed using SPSS, version 27.0 (IBM Corp). The data will be presented using the frequency, percentage table, and appropriate bar charts or graphs for the variables studied.

The spatio-temporal analysis of patterns and distribution of leptospirosis hotspot areas in Selangor will be analyzed using the Moran I and Getis-Ord Gi* spatial statistical tools in ArcGIS Pro. Meanwhile, the relationship between leptospirosis hotspot areas and the hydroclimatic hotspot areas will be analyzed using Pearson correlation. Next, a generalized linear mixed model will be used to examine the correlation between the studied variables. The best-fitted model will be determined through the multivariate analysis by equations that give the lowest value of the Akaike information criterion result.

A predictive model for leptospirosis hotspot areas will be developed using the Phyton intelligent software to run the artificial neuron network analysis. The Phyton software will be able to examine the best-fit model for the association between leptospirosis hotspot areas with its hydroclimatic indicators.

### Ethical Consideration

The study protocol obtained ethical approval with expedited review by the Medical Research and Ethics Committee, Ministry of Health Malaysia, on August 25, 2022 (NMRR ID-22-01548-C0Z). Data collection began in October 2022 and is expected to complete in March 2023.

## Results

The results of this study are expected to be published by the end of 2024. The leptospirosis distribution and clusters are expected to be significantly associated with rainfall, land surface temperature, and the river hydrometric level. Both models, constructed through the use of statistical analysis and Python intelligent software, possess the potential to offer a comprehensive and multidimensional assessment of leptospirosis distribution and clustering. This integrated approach may enhance the comprehension of disease dynamics and facilitate the development of more effective strategies for prevention and control.

## Discussion

### Principal Findings

Globally, leptospirosis occurs in humid and tropical regions favoring the dwelling of mammals, including rodents, the main reservoirs of *Leptospira* causing the disease [6]. The threat of leptospirosis as an emerging neglected zoonotic disease is due to world climate changes and globalization. Flooding, rising temperatures, and the concurrent increase in rodent density greatly influence its distribution [5]. Domestic animals may harbor the organisms, but rodents are the main reservoirs of leptospires that incidentally transmit infections to humans through contact with contaminated water soiled with the reservoirs’ urine [31].

Most infections with the pathogenic *Leptospira* cause subclinical manifestations or remain asymptomatic [1]. Symptomatic patients can have clinical manifestations, from mild fever, chills, headache, and muscle tenderness to severe forms characterized by dysfunction of multiple organs, including the liver, lungs, kidneys, and brain [1,32]. Commonly noticed manifestations are muscle pain involving the lower back and calves as well as conjunctival suffusion [32]. Severe forms of the disease are found in 10% of affected individuals who develop life-threatening pulmonary hemorrhages, Weil syndrome, and acute kidney injury [1].

The influence of climate change, such as the variations in rainfall, land temperature, and frequent floods, increases leptospirosis cases [5]. Studies in South America and East Asia ascertained the association of these factors to the disease, where heavy rainfall and flood events during the El Nino phenomenon and rainfall events that cause an increase in soil moisture impact regional leptospirosis [16,33]. A local study using weather data and artificial neuron network modeling to link leptospirosis occurrence in Seremban discovered their significant correlation with temperature and rainfall at a lag between 12 and 20 weeks [29].

Targeting hotspot areas for disease control is one of the widely used strategies. A hotspot can be described as an area that shows evidence of increased disease incidence, a higher risk of transmission, or a greater chance of disease emergence [34]. Many environmental factors influence leptospirosis incidence. They include factors involving occupation, natural disasters, and recreational or avocational exposure [9]. This study will focus on the major hydroclimatic factors for leptospirosis, including rainfall, river hydrometric level, and land surface temperature, due to global climate change dynamics and concurrent increase in disease incidence [16,18,28].

### Strengths

This research will act as a pilot study for the whole country for a spatio-temporal analysis of leptospirosis with its association with the hydroclimatic factors. The predictive model developed could be used to replicate and improve similar studies in the future by investigating other hydroclimatic factors, such as water storage in the ground and in vegetation.

### Limitations

The limitation of the study is that it only evaluates 3 main hydroclimatic factors in Malaysia that might only be applicable to regions or countries with the same climate. There may be additional hydrological factors that contribute to leptospirosis hotspot areas. Furthermore, this study did not analyze other significant environmental or nonenvironmental factors that might contribute to leptospirosis hotspot areas.

### Conclusion

In conclusion, this study may illuminate whether leptospirosis infections occur in clusters and the role of hydroclimatic factors in contributing to the emergence of leptospirosis hotspot areas in Selangor. Moreover, public health managers can use the findings from the hotspot map and statistical models, along with the Python intelligent software analysis, to identify areas of high disease prevalence and implement targeted interventions to prevent and control the spread of leptospirosis more effectively.

## Appendix

Multimedia Appendix 1 Peer-review reports from the Jawatankuasa Penilaian Permohonan Geran Putra Berfokus Jabatan Kesihatan Komuniti (GP-F/JKK) (Malaysia).

## Abbreviations

DID: Department of Drainage and Irrigation
JUPEM: Department of Survey and Mapping
LST: land surface temperature
SEA: South East Asia

## Data Availability

Data availability can be accessed on the NASA EarthData website for satellite images and the DID for hydrological data on river hydrometric levels. Data were also made available from respective agencies with requests and approval letters.
