# Supplementary material for: Spatio-Temporal Analysis of Leptospirosis Hotspot Areas and Its Association With Hydroclimatic Factors in Selangor, Malaysia: Protocol for an Ecological Cross-sectional Study
Source: JMIR Res Protoc. 2023 May 15;12:e43712. doi: 10.2196/43712 (PMC10227704; doi:10.2196/43712)
Supplement: Multimedia Appendix 1 [file resprot_v12i1e43712_app1.pdf]

UPM<br>UNIVERSITI PUTRA MALAYSIA<br>BERILMU BERBAKTI	**GERAN POTRA BERFOKUS JABATAN KESIHATAN KOMUNITI (GP-F/JKK)**
	**FORM OF REVISIONS**

Response to Reviewer's Comments

No	Comments	Response	Page
**1**	*Justification of 'selected disease link to climate' need to be strengen*	The rationale for leptospirosis linked with the environmental factors studied in this research (the hydroclimatic factors) was explained in the paragraphs in the introduction and literature review chapters.<br><br>The following paragraphs were added to the Grant Application Form:<br>1.3 Problem statements,<br>1.4 Significance of study<br>1.5 Leptospirosis disease<br>1.6 The transmission cycle<br>1.7 Leptospirosis hotspot	**9, 10, and 11**
**2**	*The factors that contributes to Leptospirosis are many e.g. student central, sanitation level, food hygiene, waste diagnosed – which seem to be more important to study*	The reason for the factors mentioned studied in the research was explained in the problem statement section.<br><br>Based on the epidemiological triad, this research focuses on environmental factors. Besides, this study utilises tools that analyses climate factors that include rainfall and temperature from satellite data and river hydrometric levels data from the Department of Irrigation and Drainage (DID).<br><br>Other factors that contributes to leptospirosis incidence were not included in this research. This limitation would be an opportunity for future researchers to explore.	**7 and 8**
**3**	*The analysis seems to be very complex and not actually taught in any of our Dept's courses.*	Analysis of data obtained will be processed with the supervision of supervisor and co-supervisors from the Department of Community Health (JKK) and the Department of Electrical & Electronic Engineering.<br><br>Statistical analysis of Moran's I, Getis-Ord Gi and GLMM using ArcGIS and SPSS Softwares, will be	**21 and 22**

		further studied from external lectures with the supervision of supervisors from JKK.<br><br>The analysis using the Python Software running the ANN analysis and modelling to develop the leptospirosis predictive model, will also be further studied from external lectures with the supervision of a supervisor from the Department of Electrical & Electronic Engineering.<br><br>Since the methodology and analysis using remote sensing and ANN analysis is relatively new in the public health field, it will be a pleasure to be shared with the department in the JKK's CME session and other occasions.	
**4**	*'similarity test' with method previous DrPH student research*	To the best of the researcher's knowledge, this topic and methodology has never been used by DrPH candidate from the JKK Department, UPM.<br><br>Using remote sensing data in public health research would be a new field to explore in public health. Future researchers could also apply artificial intelligence analysis to predict events that may be of public health concern and importance.	
**5**	*To address the weakness mentioned*	The weaknesses mentioned were addressed and discussed with the research team. Each weakness was addressed with the required actions and corrections.	-
**6**	*Students need to show he understands the analysis (method & results) by explaining step by step of how they are done*	Knowledge of the theories and application of the analysis will be gained from the supervisory committee and external sources, such as attending required courses.	-
**7**	*Title & first impression – Grammatically, somehow not quite right; suggest – 'Predictive model of spatio-temporal analysis….'*	The research's general objective is to examine the spatio-temporal distribution of leptospirosis hotspot areas using GIS and remote sensing techniques. Thus the title begins with "Spatio-temporal analysis ".<br>In addition, the research team has agreed on the research title. The research also obtained ethical approval from the NMRR on August 25, 2022.	**1**

Reviewed by, [signature] 10/11/2022

ASSOC. PROF. DR. ROSLIZA ABDUL MANAF
Public Health Medicine Specialist
Department of Community Health
Faculty of Medicine & Health Sciences
Universiti Putra Malaysia
MMC No. 33944

**PENGESAHAN PENILAI**<br>**ENDORSEMENT BY THE EVALUATOR**	Pilih Cadangan Perakuan&<br>Taraf Bintang
Cadangan Projek (sila pilih satu sahaja)<br><br>*Project Recommendation (Please select one only)*<br>*1- Disokong sepenuhnya tanpa pindaan (Highly rescommended)*<br>*2- Perlu pinddan dan dinilai semula (Resubmit with corrections)*<br>*3- Tidak disokong (Not Recommended)*	Pilih cadangan perakuan<br>*Please select recommendation*

Tarikh<br>*Date*	
Tandatangan Panel Penilai<br>*Evaluatorss' Signiture*	
Nama Penilai & Cop<br>*Name of Evaluator & Official Stamp*	
No. Telefon Pintar/<br>No. Tel Bimbit<br>*Office Telephone Number/ Handphone Number*	
Emel<br>*E-mail*	
